# Fly model causes neurological rethink

**DOI:** 10.7554/eLife.01820

**Published:** 2013-12-11

**Authors:** Madhumala K Sadanandappa, Mani Ramaswami

**Affiliations:** 1 **Madhumala K Sadanandappa** is in the National Centre for Biological Sciences-TIFR, Bangalore, Indiamadhumala@ncbs.res.in; 2 **Mani Ramaswami** is an *eLife* Reviewing Editor, and is in the School of Genetics and Microbiology, School of Natural Sciences, Smurfit Institute of Genetics and Trinity College Institute of Neuroscience, Trinity College Dublin, Dublin, Ireland and in the National Centre for Biological Sciences-TIFR, Bangalore, Indiamani.ramaswami@tcd.ie

**Keywords:** endosome, neuropathy, genetics, synapse, animal models of disease, D. melanogaster

## Abstract

A *Drosophila* model for a neurological disorder called type 2B Charcot-Marie-Tooth disease reveals that it has its origins in a partial loss of function, rather than a gain of function, which points to the need for a new therapeutic approach.

**Related research article** Cherry S, Jin EJ, Özel MN, Lu Z, Agi E, Wang D, Jung W-H, Epstein D, Meinertzhagen IA, Chan C-C, Hiesinger PR. 2013. Charcot-Marie-Tooth 2B mutations in *rab7* cause dosage-dependent neurodegeneration due to partial loss of function. *eLife* **2**:e01064. doi: 10.7554/eLife.01064

**Image** A fly model has provided fresh insights into a disorder that affects the peripheral nervous system

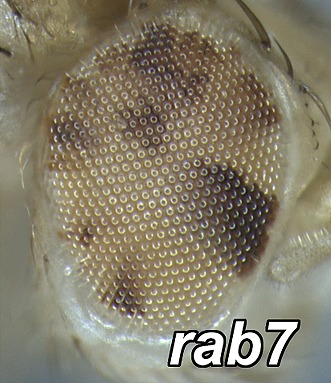

While human genetics is essential for the discovery of new genes associated with inherited diseases, studies in animal models are critical for understanding the underlying mechanisms and pathways. In general, mammalian or vertebrate models are believed to better mimic human disease pathologies, but invertebrate models—such as *Drosophila*, *C. elegans* and even yeast—remain extraordinarily useful and can, in some cases, provide insights that lead to new approaches to therapies. Now, in *eLife*, researchers at the University of Texas Southwestern Medical Center (UTSW), National Taiwan University (NTU) and Dalhousie University report that they have used a *Drosophila* model to obtain results that could lead to new treatments for a group of disorders that affect the peripheral nervous system (Cherry et al., 2013).

Charcot-Marie-Tooth disease was first described in the late 1880s by Jean-Martin Charcot, Pierre Marie and Howard Henry Tooth, and we now know that it is caused by mutations in any of more than 30 genes. Charcot-Marie-Tooth disease is clinically subdivided into type 1, which begins in childhood and involves the loss of the myelin sheaths around sensory and motor axons, and type 2, which can begin at any age and involves axon degeneration. Remarkably, most of the mutations that lead to Charcot-Marie-Tooth disease show a dominant pattern of inheritance (Barisic et al., 2008).

Type 2B Charcot-Marie-Tooth disease (CMT2B) is caused by mutations in a residue of *rab7A*, a gene that encodes a small enzyme that is involved in regulating endosomes and lysosomes in all cells (Kwon et al., 1995; Elliott et al., 1997; Verhoeven et al., 2003). Biochemical and structural studies previously suggested that these mutations decrease the affinity of the Rab7A enzyme for GDP, which eventually leads to increased interactions between the enzymes and effector proteins (McCray et al., 2010). Furthermore, studies in different cell lines demonstrated that over-expression of the Rab7 mutants alters a number of signalling pathways and leads to increased interactions between Rab7 and peripherin, a protein that is found in neurons (Spinosa et al., 2008; Basuray et al., 2013; Zhang et al., 2013). Together, these studies suggest a model in which CMT2B disease phenotypes arise from an enhanced function of Rab7A (McCray et al., 2010), which implies that inhibiting the Rab7A pathway could be potentially useful for managing the progress of the disease (Figure 1). However, this might not be the full story.

**Figure 1.**
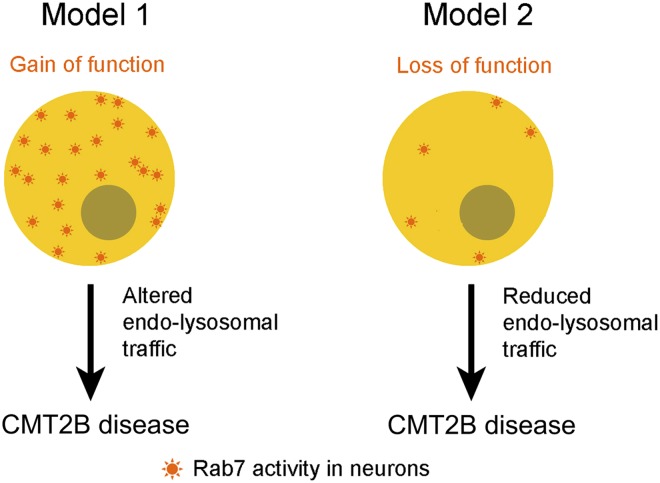
Two models for linking mutations in the *rab7A* gene to type 2B Charcot-Marie-Tooth (CMT2B) disease. Some researchers have argued that the disease is caused by the mutations leading to enhanced Rab7A activity (Model 1), which suggests that the disease could be treated by inhibiting the Rab7A pathway. However, Cherry, Jin et al. propose that the primary cause of the disease is a lack of Rab7A activity (Model 2), which suggests that patients with CMT2B disease should be given treatments that stimulate rather than inhibit the Rab7 pathway.

Now Robin Hiesinger (UTSW), Chih-Chiang Chan (NTU) and co-workers—including Smita Cherry and Jennifer Jin (both UTSW) as joint first authors—have developed the first animal model for CMT2B. They created a null allele of the *rab7* gene in *Drosophila,* as well as inducible transgenes expressing the following variants: wild-type, constitutively active, dominant-negative and four mutants relevant to CMT2B, along with wild-type and one mutant for human *rab7A*. They assessed the effects of genetic perturbations, primarily in photoreceptor cells, which are not required for animal survival.

An immediate surprise was that *rab7* null mutant photoreceptors showed no obvious developmental defects, but instead showed phenotypes that were reminiscent of a sensory neuropathy (including age-dependent and activity-dependent synaptic loss, and also progressive degeneration of photoreceptor neurons). In sharp contrast, flies expressing the constitutively active form of the Rab7 enzyme showed normal synaptic function, normal eye morphology and photoreceptor integrity. These observations strongly (and unexpectedly) suggest that disease-relevant CMT2B phenotypes may arise from Rab7 loss of function.

To further test this idea, Cherry, Jin et al. investigated if over-expression of the mutant forms of Rab7 had any effect on wild type Rab7 function in *Drosophila* peripheral neurons. They found no evidence for any of the symptoms of CMT2B. Moreover, these mutations appeared to retain significant levels of wild-type function, as evidenced by the fact that their expression allowed *rab7* null mutants, which normally do not survive, to live well into adulthood. The conclusion that CMT2B—and, possibly, other neurological disorders—arises from a partial loss of Rab7 function is also supported by the finding that flies that are heterozygous for the *rab7* null mutation also show light-induced progressive eye degeneration.

It is interesting that the observations of Cherry, Jin et al. are not inconsistent with several previous observations in mammalian cells, such as the finding that CMT2B mutant proteins can provide essential, native Rab7 functions (Spinosa et al., 2008; McCray et al., 2010). In addition, through a detailed structural and biochemical analysis, some researchers have argued against a novel toxic gain-of-function associated with CMT2B disease-variant proteins (McCray et al., 2010). Now, newly equipped with in vivo analyses in *Drosophila*, Cherry, Jin et al. reinterpret several of these observations as supporting a loss-of-Rab7 origin for CMT2B.

Phenotypes in *Drosophila*, as well as the underlying molecular networks, need not be entirely congruent with human disease. In this case, it is possible that the mutant Rab7 proteins affect mammalian cells and *Drosophila* cells in different ways. Indeed, *Drosophila* heterozygotes for *rab7* are mostly healthy, but human heterozygotes for CMT2B mutations show disease. Possible explanations for this include differences in lifespan and/or the levels of gene expression. However, taken together, this new analysis provides a strong evidence to support the notion that CMT2B disease pathologies arise from the partial loss of Rab7 function. This new work forces a re-examination of the mechanistic basis for CMT2B and suggests that patients may need to have the activity of the Rab7 pathway stimulated rather than reduced.
