# Correction: Fly model causes neurological rethink

**DOI:** 10.7554/eLife.02094

**Published:** 2013-12-20

**Authors:** Madhumala K Sadanandappa, Mani Ramaswami

Sadanandappa MK, Ramaswami M. 2013. Fly model causes neurological rethink. *eLife* **2**:e01820. doi: http://dx.doi.org/10.7554/eLife.01820. Published 11 December 2013

Madhumala K Sadanandappa’s last name was incorrectly spelled ‘Sadandappa’.

The article has been corrected accordingly.

